# A new family of carbonaceous cathodes for rechargeable batteries through electronic structure tuning engineering

**DOI:** 10.1093/nsr/nwaa185

**Published:** 2020-09-02

**Authors:** Michel Armand

**Affiliations:** Centre for Cooperative Research on Alternative Energies, Basque Research and Technology Alliance, Spain

Electric vehicles (EVs) and grid storage for renewable energies will require batteries in tens of millions of tons, and thus they need to be sustainable. EVs call also for batteries with higher energy density than currently available, and this value is intrinsically limited by the properties of the transition metal oxide (TMO) cathodes [1]. There are two possible strategies to overcome these limitations: i) increase the operating voltage in the >4.4 V range; and ii) attempt to use more than one electron per transition metal [2]. The first strategy faces problems of electrolyte stability limit and oxygen evolution, and thus safety hazards. The second is limited to vanadium and nickel, but using only 1.3 electrons for the former, and the latter must be diluted in 300% manganese lattice. Cobalt, used today, nickel and vanadium are metals for which shortages are foreseen with the expansion of the battery market. There have been countless research efforts over the past 30 years, but, to date, the current TMO cathode has practically reached its limit in terms of its theoretical specific capacity and voltage performance.

Carbon, as graphite, in a battery is synonymous to anode material because of its low electrochemical potential (<0.5 V vs. Li/Li^+^), determined by the high energy level *sp*^2^-*p*_z_ orbital in the form of a weak π bond. An open question is whether the electrochemical potential of carbonaceous materials can be tuned to a level comparable to that of the current widely used TMO cathodes. In the 1970s, we introduced TM salts with high valence state into graphite, e.g. graphite chromium oxide (C_8_CrO_3_), which could effectively ‘pump’ the carbon electrons into the ‘d’ shells of TM and therefore improve the redox potential of graphite-based electrodes. The Na | *β*-Al_2_O_3_ | C_8_CrO_3_ cell exhibited a surprisingly high voltage of 3.9 V, but the redox reactions were still pinned to the ‘d’ orbitals of the TM [3].

Recently, Prof. Chuying Ouyang's group from Jiangxi Normal University and Prof. Siqi Shi's group from Shanghai University achieved breakthroughs in the design of carbonaceous materials as cathodes for rechargeable LIBs/SIBs [4]. Amazingly, they showed that electronic structure engineering via a p-type doping strategy can tune the potential of graphite derivatives to reach the requirements for cathode application (2.7–3.7 V), leading to a record-breaking high energy density (>1000 Wh kg^−1^) (Fig. 1). This is done resorting only to light elements of the first row, as in CBF_2_—an example of a host material devoid of any TM.

**Figure 1.**
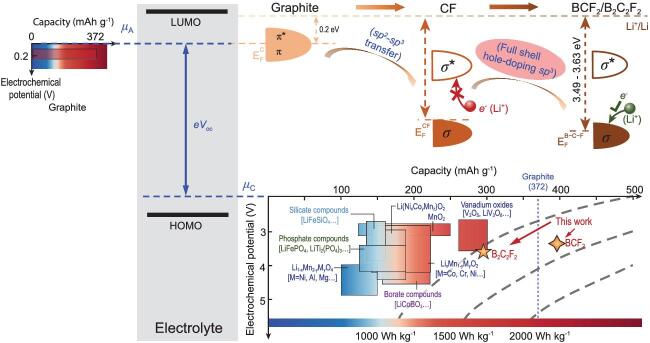
Schematic open-circuit voltage (V_oc_) of a battery. The energy separation of the lowest-unoccupied-molecular-orbital (LUMO) and the highest-occupied-molecular-orbital (HOMO) is the electrolyte window. Electrochemical potential vs. capacity is presented for both graphite-anode and cathodes. The cathodes are commonly transition-metal (TM) compounds, which have layered, spinel or olivine crystal structures. The figure is from [4].

This is a new paradigm for battery design, which could address issues related to the battery energy-density limit as well as transition-metal cost and shortages. In a broader sense, the success of the full shell *p*-doping strategy to shift-down the Fermi-level of graphite may motivate more researchers to evaluate the link between the electrochemical potential and the band structure engineering of electrodes, which could help to guide rational design of these compounds in the future and inform prospective theoretical and experimental research in this field.

## FUNDING

This work was supported by the Basque Government through the ELKARTEK-2016 program.

***Conflict of interest statement***. None declared.

## References

[1] Armand M , Tarascon JM. Nature 2008; 451: 652–7. 10.1038/451652a 18256660

[2] Kang B , Ceder G. Nature 2009; 458: 190–3. 10.1038/nature07853 19279634

[3] Armand M , Touzain P. Mater Sci Eng 1977; 31: 319–29. 10.1016/0025-5416(77)90052-0

[4] Wang Z , Wang D, Zou Z et al. Natl Sci Rev 2020; 7: 1768–75. 10.1093/nsr/nwaa174 PMC8288616 34691510

